# Gait Correlation Analysis Based Human Identification

**DOI:** 10.1155/2014/168275

**Published:** 2014-01-29

**Authors:** Jinyan Chen

**Affiliations:** School of Computer Software, Tianjin University, Tianjin 300072, China

## Abstract

Human gait identification aims to identify people by a sequence of walking images. Comparing with fingerprint or iris based identification, the most important advantage of gait identification is that it can be done at a distance. In this paper, silhouette correlation analysis based human identification approach is proposed. By background subtracting algorithm, the moving silhouette figure can be extracted from the walking images sequence. Every pixel in the silhouette has three dimensions: horizontal axis (*x*), vertical axis (*y*), and temporal axis (*t*). By moving every pixel in the silhouette image along these three dimensions, we can get a new silhouette. The correlation result between the original silhouette and the new one can be used as the raw feature of human gait. Discrete Fourier transform is used to extract features from this correlation result. Then, these features are normalized to minimize the affection of noise. Primary component analysis method is used to reduce the features' dimensions. Experiment based on CASIA database shows that this method has an encouraging recognition performance.

## 1. Introduction

Biometrics is a technology that makes use of the physiological or behavioral characteristics to authenticate or identify people [1]. The most commonly used biometrics applications are fingerprint and iris based identification.

Human gait was firstly studied in the medical field [2–5]. Doctors analyzed human gait to find out whether patients had health problem. Later, researchers [5] found that just like fingerprint and iris, almost everyone had his distinctive walking style. So someone believed that gait could also be used as a biological feature to identify a person. Comparing to fingerprint and iris based identification, human gait identification has the following advantages [6]: (1) it does not require the user's interaction and (2) it can be done at a distance, as long as the gait is visible.

Current human gait identification can be divided into two categories: model-based methods and motion-based methods.

Model-based approaches aim to describe human movement using a mathematical model, for example, Cunado et al. [7] used Hough translation to extract arms, legs, and torso and use articulated pendulum to match the moving body parts. Yoo et al. [8] divide the body into head, neck, waist, leg, and arm by image segmentation and then obtained the moving curve of these body parts. Lee and Grimson [9] applied 7 ellipses to model the human body and applied the ellipses' movement features to identify human. Yam et al. [10, 11] used dynamically coupled oscillator to describe and analyze the walking and running style of a person. Tafazzoli and Safabakhsh [12] constructed movements model based on anatomical proportions then Fourier transform was used to analysis human walking style. Dupuis et al. [13] created probabilistic based gait modeling to describe the human walking.

Motion-based methods consider the human gait as a sequence of image and extract features from these images. Cheng et al. [14] employed Hidden Markov Models to analyze the relationship among these images. Chen et al. [15] used parallel HMM to describe the features of human gait. Yu et al. [16] overlapped all the images to get Gait Energy Image (GEI) and GEI was used as the features to identify humans. Similar to GEI, Fan et al. [17] took Chrono-Gait Image as the gait features. Kale et al. [18] used “frieze” patterns to extract features from image sequence and used these features to identify humans. Liu et al. [19] represent one's walking style by outermost contour.

The raw features extracted by motion-based methods usually contained several cures or images, so Gabor transform [20], Random Forest algorithm [12], and Fourier transform [21, 22] were used to reduce the dimensions of these features.

### 1.1. Overview of Our Approach

**Figure 1:**
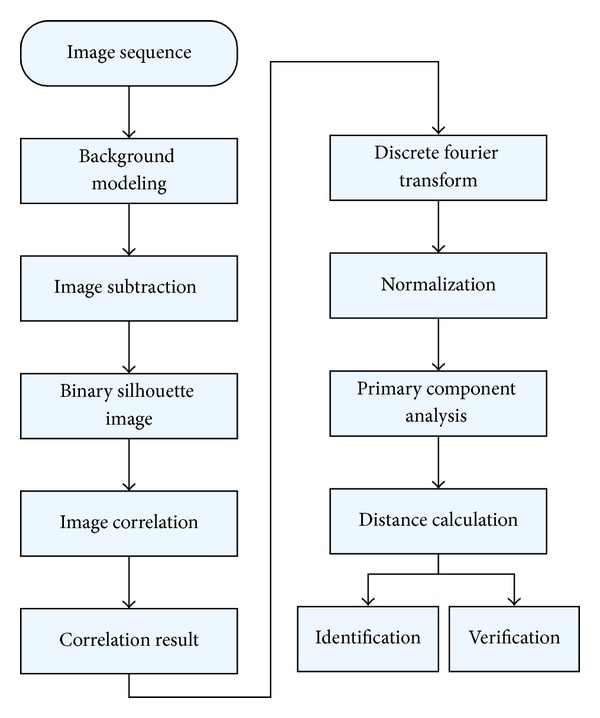
Overview of our human identification method.

Unlike traditional image correlation based identification [16, 23, 24], this paper aims to build a human gait identification method not only in spatial domain but also in temporal domain. A median model is used to estimate the background. By background subtraction, we get the binary human silhouette. Every silhouette is correlated with its neighbor at horizontal, vertical, and temporal axis. The size of the correlation result is used to construct the walking feature curves and every feature curve consists of several walking cycles. The feature curve is normalized to build a standardized curve. For every image sequence, we can get totally 13 standardized curves. Primary component analysis is used to extract features from these above curves. The overview of the proposed algorithm is shown in Figure 1.

### 1.2. Our Contribution

The main purpose and contributions of this paper can be summarized as follows.

1. We extract 13 feature curves from the human gait images sequence. These 13 curves described the feature of human walking.
2. We proposed a method to construct normalized human gait feature vectors. PCA is used to reduce the dimensions of gait feature vectors.
3. We discuss the relationship between accuracy of identification and the shift length along horizontal axis and vertical axis and try to find out the best shift length.

## 2. Feature Extraction

### 2.1. Silhouettes Sequence Extraction

In this paper, the human gait is considered as a binary image. Gauss background model is used to estimate the background. We use the method proposed by Wang et al. [25] to subtract the background from the foreground of the image as follows:

(1) $$\begin{array}{c} D\left(a,b\right)=1-\frac{2\sqrt{\left(a+1\right)\left(b+1\right)}}{\left(a+1\right)+\left(b+1\right)}\cdot \frac{2\sqrt{\left(256-a\right)\left(256-b\right)}}{\left(256-a\right)+\left(256-b\right)}, \end{array}$$

*D*(*a*, *b*) is the difference between point *a* (pixel in foreground image) and point *b* (pixel in background image).

### 2.2. About the Image Sequence Correlation

Image correlation is a shift-invariant feature method and was first proposed by Otsu and Kurita [26]. Let *f*(*R*) be *D*-dimensions data with *R* = (*x*_1_, *x*_2_,…, *x*_*D*_); then the correlation function is defined as

(2) $$\begin{array}{c} \psi \left(f\left(R\right),S_{i}\right)=\int f\left(R\right)f\left(R+S_{i}\right)dR, \end{array}$$

where (*S*_*i*,1_,…, *S*_*i*,*D*_) is the shift vector.

**Figure 2:**
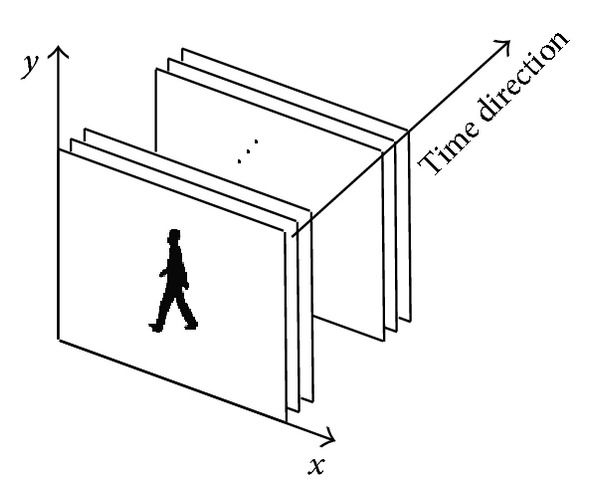
Three dimensions of the original image.

**Figure 3:**
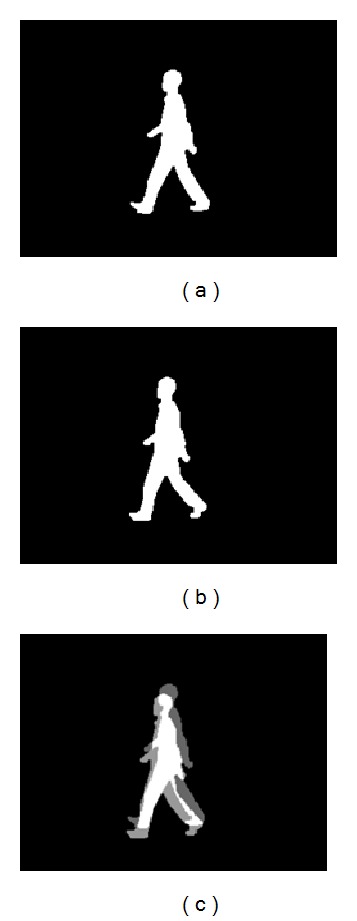
(a) An image whose frame index is *t*. (b) An image whose frame index is *t* + 1. (c) The image expression of correlation result (the bright area) of image *t* and image *t* + 1, *S*_*i*_ = (0,5, 1).

Given a human gait image sequence, *f*(*R*_*t*_) means an image in the sequence whose frame index is *t*. If *S*_*i*_ = (*s*_*i*,*x*_, *s*_*i*,*y*_, *s*_*i*,*t*_), *f*(*R*_*t*_ + *S*_*i*_) means we get the frame whose frame index is *t* + *s*_*i*,*x*_, then shift its *s*_*i*,*x*_ pixels along *x*-axis and *s*_*i*,*y*_ pixels along *y*-axis. The three dimensions of the image are shown in Figure 2. The original image is shown in Figures 3(a) and 3(b) and the correlation result is shown in Figure 3(c).

Given a human walking cycle, images sequence consists of *T* frames, the features of this human gait images sequence can be described as

(3) $$\begin{array}{c} \Omega =\left(\begin{array}{c} \Psi \left(f\left(R_{1},S_{1}\right),\ldots ,\Psi \left(f\left(R_{T-1}\right),S_{1}\right)\right)\\ \Psi \left(f\left(R_{1}\right),S_{2}\right),\ldots ,\Psi \left(f\left(R_{T-1}\right),S_{2}\right)\\ \vdots \\ \Psi \left(f\left(R_{1}\right),S_{I}\right),\ldots ,\Psi \left(f\left(R_{T-1}\right),S_{I}\right) \end{array}\right). \end{array}$$

### 2.3. The Normalized Human Gait Image Correlation

**Figure 4:**
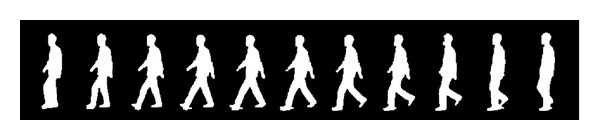
A human Gait cycle.

**Figure 5:**
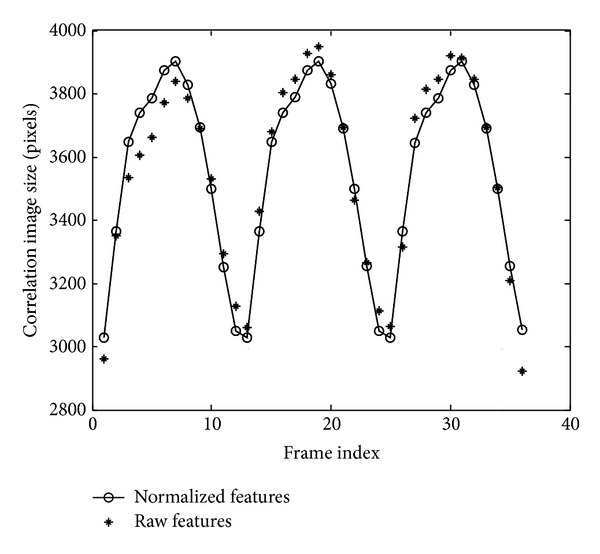
Correlation image size calculated by raw features and by normalized features.

Figure 4 shows a gait cycle. In the first stance, the person is at rest and the silhouette size is minimum, this corresponds to the valley at Figure 5; then he steps out his leg, the silhouette size will increase with his stepping out, in the sixth stand his legs and arms are completely separated and this corresponds to the maximum at Figure 5. Then, the legs and arms are closed, at stance 11 the size reaches minimum again. In fact, this is not a complete gait cycle but a “half” cycle, but the previous “half” cycle and the remaining “half” cycle are symmetrical, so in this paper we call this “half” gait cycle as one gait cycle.

Formula (3) is the matrix expression of the curves shown in Figure 3. *Ω* can be used as features to identify humans. But these features are not robust. The human silhouettes are prone to be affected by the noise and deformation. We will transform the curve into standard curve to make features more robust by the following steps.

(1) Discrete Fourier transforms: by dividing the walking images into *C* walking cycles as shown in Figure 3, we can find the start (end) of a walking cycle by finding the minimum point in Figure 5. For every walking cycle *c* = (1,2,…, *C*), if this walking cycle consists of *N*_*c*_ images, the value of *N*_*c*_ may vary slightly based on different persons or different walking cycles. We can use Discrete Fourier transform to describe the features of these points in Figure 5 as follows:
  (4) $$\begin{array}{c} x\left[n\right]=\overset{N_{c}-1}{\underset{m=0}{\sum }}\hat{x}{\left[m\right]}^{i(2\pi /N_{c})mn},\;n=0,\ldots ,N_{c}-1, \end{array}$$
  (5) $$\begin{array}{c} \hat{x}\left[m\right]=\overset{N_{c}-1}{\underset{n=0}{\sum }}x\left[n\right]e^{-i(2\pi /N_{c})mn},\;m=0,\ldots ,N_{c}-1. \end{array}$$
  *x*[*n*] is the discrete points and $\hat{x}\left[m\right]$ is the Fourier coefficients.
(2) Normalization: Considering that different walking cycles have different frame count (*N*_*c*_), we need to “align” *N*_*c*_ to a fixed value. In this paper, we divide every walking cycle into *K* parts, where *K* is a fixed value (in this paper *K* = 12). That is
  (6) $$\begin{array}{c} x^{\prime }\left[k\right]=\frac{k}{K}\left(N_{c}-1\right),\\ {\hat{x}}^{\prime }\left[m\right]=\overset{K-1}{\underset{k=0}{\sum }}x^{\prime }\left[k\right]e^{-i(2\pi /K)mk},\;m=0,\ldots ,K-1. \end{array}$$

Given person *p*, the normalized feature matrix for walking cycle *c* can be expressed as

(7) $$\begin{array}{c} \Omega \left(p,c\right)=\left(\begin{array}{c} {\hat{x}}^{\prime }{\left[1\right]}_{1},\ldots ,{\hat{x}}^{\prime }{\left[K\right]}_{1}\\ {\hat{x}}^{\prime }{\left[1\right]}_{2},\ldots ,{\hat{x}}^{\prime }{\left[K\right]}_{2}\\ \vdots \\ {\hat{x}}^{\prime }{\left[1\right]}_{I},\ldots ,{\hat{x}}^{\prime }{\left[K\right]}_{I} \end{array}\right). \end{array}$$

Given *C* walking cycles, we use the normalized *Ω*(*p*, *c*) as the final features. The final feature matrix for person *p* can be expressed as

(8) $$\begin{array}{c} \tilde{\Omega }\left(p\right)=\frac{1}{C}\overset{C}{\underset{c=1}{\sum }}\Omega \left(p,c\right). \end{array}$$

By inverse Fourier transforms (Formula (5)), we can also get the correlation image size from $\tilde{\Omega }(p)$ inversely; the correlation image size calculated by Fourier transforms is shown in Figure 5.

### 2.4. Reduce Dimensions of Features

We define $\overset{-}{\Omega }$ as follows:

(9) $$\begin{array}{c} \overset{-}{\Omega }=\frac{1}{P}\overset{P}{\underset{p=1}{\sum }}\tilde{\Omega }\left(p\right). \end{array}$$

*P* is the human count in the test dataset. That is to say, $\overset{-}{\Omega }$ is the average value of all human gait features. Then, we calculate the covariance matrix COV as follows:

(10) $$\begin{array}{c} \text{COV}=\sum \left(\tilde{\Omega }\left(p\right)-\overset{-}{\Omega }\right){\left(\tilde{\Omega }\left(p\right)-\overset{-}{\Omega }\right)}^{T}. \end{array}$$

Principal component analysis is used to reduce the dimensions of $\tilde{\Omega }\left(p\right)$. The reduced $\tilde{\Omega }\left(p\right)$ can be expressed as

(11) $$\begin{array}{c} {\tilde{\Omega }}^{\prime }\left(p\right)=\left(e_{1},e_{2},\ldots ,e_{k}\right)\tilde{\Omega }\left(p\right), \end{array}$$

where (*e*_1_, *e*_2_,…, *e*_*k*_) are the eigenvectors selected by PCA from covariance matrix COV.

### 2.5. Human Gait Identification

In the scenario of identification, the gait feature distance between human *p* and *q* can be defined as follows:

(12) $$\begin{array}{c} D\left(p,q\right)=||{\tilde{\Omega }}^{\prime }\left(p\right)-{\tilde{\Omega }}^{\prime }\left(q\right)||, \end{array}$$

where || || means the Euclidean distance.

This probe *p* is assigned to person *k* by using nearest neighbor method. That is

(13) $$\begin{array}{c} D\left(p,k\right)=\underset{k}{\min}\;D\left(p-k\right). \end{array}$$

In the scenario of verification, the similarity between two feature matrixes is defined as the negative of distance, that is

(14) $$\begin{array}{c} \text{Sim}\left(p,q\right)=-D\left(p,q\right). \end{array}$$

In this paper, the similarity between a probe *p* and *q*_*i*_ in the gallery is defined as *z*-normed similarity [27] as follows:

(15) $$\begin{array}{c} \text{Sim}\left(p,q_{i}\right)=\frac{\text{Sim}\left(p,q_{i}\right)-{\text{Mean}}_{i}\text{Sim}\left(p,q_{i}\right)}{\text{s}.\text{d}._{i}\;\text{Sim}\left(p,q_{i}\right)}, \end{array}$$

where s.d. is standard deviation.

FAR (False Acceptance Rate), FRR (False Rejection Rate), and EER (Equal Error Rate) are used to evaluate the performance of verification [27].

## 3. Experiment and Discussion

We use the CASIA database (dataset B) [28] to evaluate our method. There are total 124 people in dataset B. For every person, there are at least 3 walking sequences captured from 12 view angles. Every walking sequence consisted of 3-4 walking cycles. In this paper, most of the experiments are based on 90° view angle except with declaration. We randomly select 2 walking sequences as the training data to get the normalized human gait vector. Then we use the remained walking sequence to evaluate performance. In this paper, for the consideration of simplicity, *s*_*i*,*x*_ has only three values: {−Δ*x*, 0, −Δ*x*}, *s*_*i*,*y*_ has three values: {−Δ*y*, 0, −Δ*y*}, and *s*_*i*,*t*_ has two values: {0, Δ*t*}. Then, we can totally get 3 × 3 × 2 = 18*S*_*i*_, *i* = 1,…, 18. Using these shift vectors *S*_*i*_, we totally get 18 feature vectors for one human gait images sequence. By using PCA, we reduce these 18 vectors to 3 vectors.

### 3.1. Features Extracted by Image Correlation

**Figure 6:**
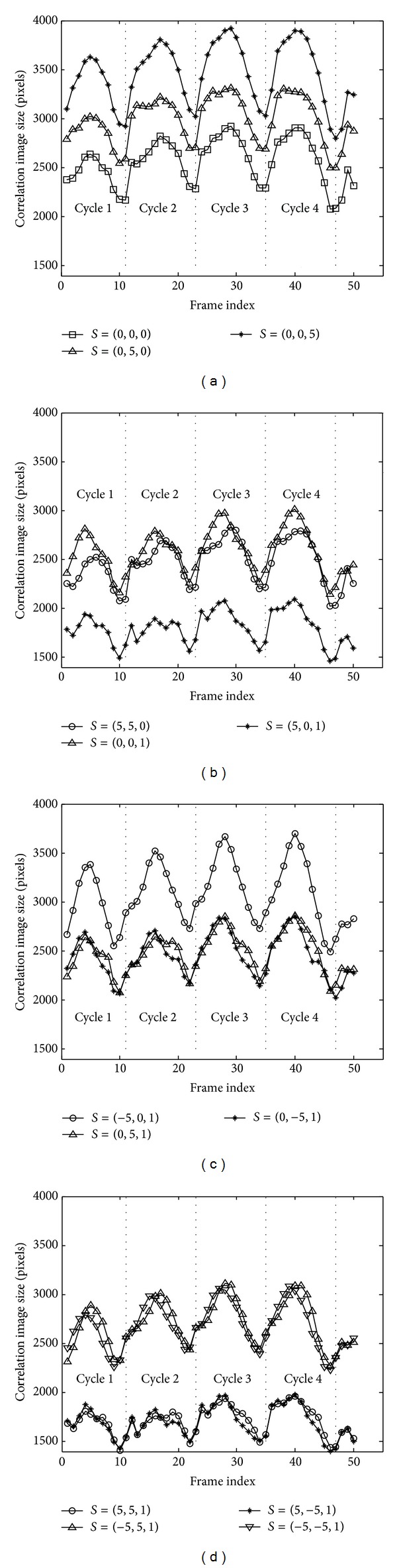
The 13 feature curves (Δ*x* = 5, Δ*y* = 5, Δ*t* = 1).

For a human gait image sequence (consisting of about four waking cycles), the 13 features curves are shown in Figure 6  (Δ*x* = 5, Δ*y* = 5, Δ*t* = 1).

### 3.2. The Result of Dimension Reduction

**Figure 7:**
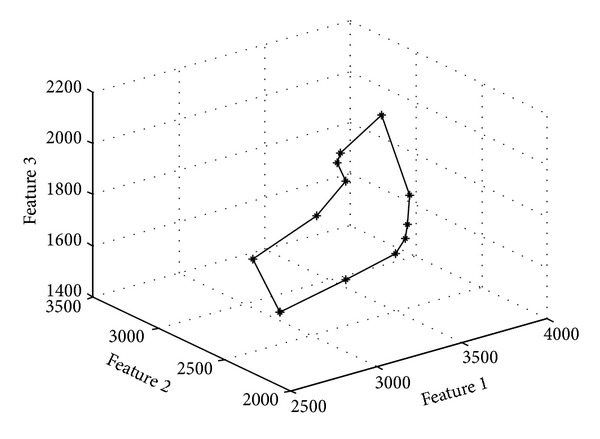
Compressed features by PCA.

From Figure 6, we can see that the 13 image correlation curves have some similarity. That is to say that there is some redundant information. We use formula (11) to reduce the dimension. We set threshold = 90% and compress the 13 human gait feature vectors into 3 vectors.  Figure 7 shows these three vectors for one human walking cycle.

**Figure 8:**
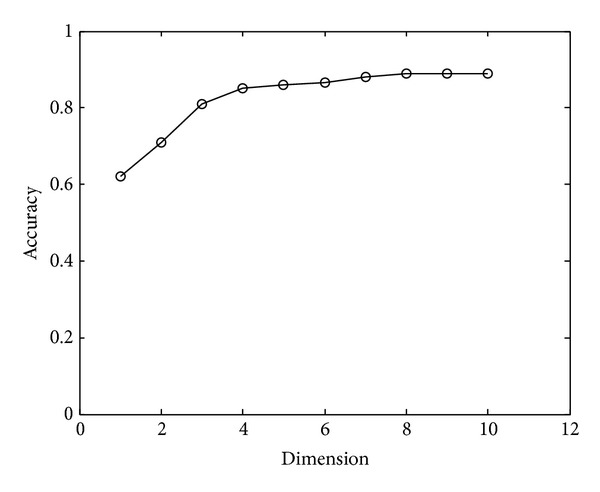
The relationship between *k* (dimensions) and top 1 recognition accuracy.

We also compared the recognition accuracy result using different dimensions (that is the *k* in Formula (11)). The experiment result is showed in Figure 8.

From Figure 8, we can see that the recognition result will reach its maximum at *k* = 4.

### 3.3. The Affection of Shift Length

From the definition of image correlation, we can see that the shift length (Δ*x*, Δ*y*, Δ*t*) along these three axes affects the correlation result. The shift length along the horizontal and vertical axes should follow below principles: the features distance between two gaits images sequences calculated by these shifts should have the maximum value. That is to say, all samples should have the maximum standard deviation as follows:

(16) $$\begin{array}{c} \Delta x=\underset{\Delta x}{\arg\;\max}\sqrt{\frac{\sum_{P=1}^{P}\left(\tilde{\Omega }\left(p\right)-\overset{-}{\Omega }\right)}{P-1}},\\ \Delta y=\underset{\Delta y}{\arg\;\max}\sqrt{\frac{\sum_{P=1}^{P}\left(\tilde{\Omega }\left(p\right)-\overset{-}{\Omega }\right)}{P-1}}. \end{array}$$

**Figure 9:**
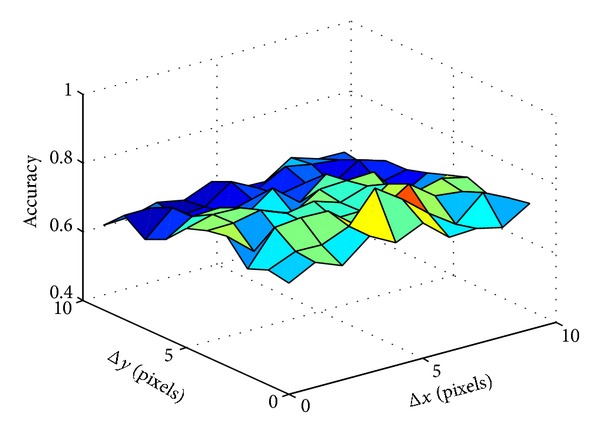
The relationship between shift length and the standard deviation.

**Figure 10:**
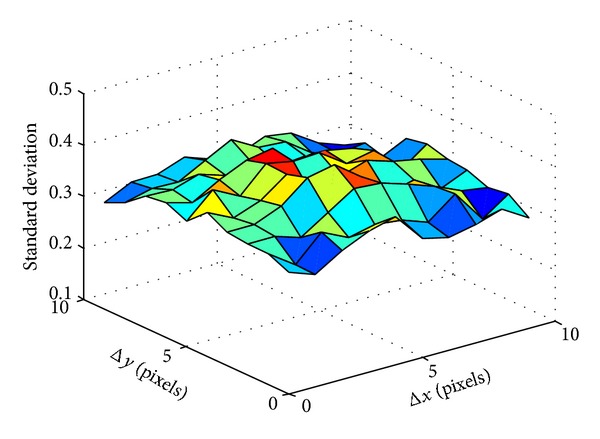
The relationship between accuracy and shift length along *x*- and *y*-axis.

To find the optimized shift length, we vary shift length along *x*- and *y*-axis from 1 to 10 pixels and calculate the standard deviation. To eliminate the affection of silhouette size, we divide the standard deviation by silhouette size. The calculation result is shown in Figure 9. The accuracy of reorganization is showed in Figure 10.

**Figure 11:**
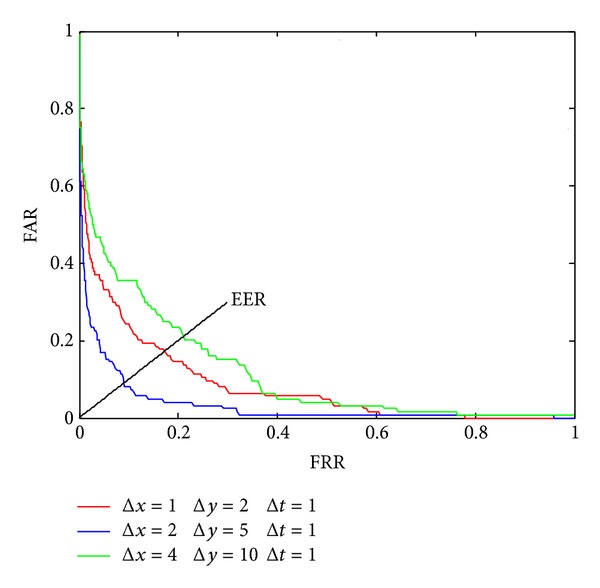
The ROC curves under different shift lengths along *x*- and *y*-axis.

For completeness, we also estimate FAR (False Acceptance Rate) and FRR (False Rejection Rate) in verification mode. The ROC (Receiver Operating Characteristic) curves are shown in Figure 11.

Comparing Figures 9, 10, and 11, we can see that both the accuracy of reorganization and ERR (Equal Error Rate) will reach optimized value at approximately the maximum point of standard derivation in Figure 9. In fact, the 5 pixels length is just the moving length along *x*-axis between two frames and the 2 pixels length is just the average moving pixels along *x*-axis and *y*-axis between two frames.

### 3.4. The Affection of View Angle

As we know, the view angle may affect the recognition accuracy, so we compare the performance under differential view angle.  Table 1 shows the experiment result.

From Table 1, we can see that our method has better performance under view angles 0, 90, and 180. That may be because the image token from above the angle is not prone to be affected by deformation.

### 3.5. Discussions and Future Works

The correlation of two images in temporal domain and spatial domain produces a new image. This image contains much information about the moving object, especially the dynamic features of the human gait. In this paper, only the areas of the correlated images are is used as the features for identification. In the future, we should try to use other features about the correlation images, for example, the texture features about the correlation images, or features from projecting the correlation images to *x*- or *y*-axis. In this paper, we only talk about two images correlation. Maybe the correlation among three or more images can also be used in the human gait identification. We will also try this in the future.

## 4. Conclusions

With the increasing demand of security control, human gait based identification will attract more interest. In the future, gait based human identification might finally become applicable just as fingerprint and iris.

The kernel idea of this paper is based on the analysis of the image correlation. Fourier transform is used to get the features from the image correlation. We discuss the relationship between shift length and the identification accuracy. We also use CASIA gait database to validate our method. The maximum classification accuracy is nearly 90% and EER is approximately 10%.

## Figures and Tables

**Table 1:** Comparison of identification performance under different view angles.

View angle	Accuracy	
	Rank 1	Rank 5
0°	65%	90%
18°	51%	66%
36°	30%	51%
54°	33%	50%
72°	62%	79%
90°	82%	92%
108°	71%	88%
126°	38%	69%
144°	39%	58%
162°	44%	69%
180°	83%	89%
